# Regulation of IkappaB Protein Expression by Early Gestation in the Thymus of Ewes

**DOI:** 10.3390/vetsci10070462

**Published:** 2023-07-13

**Authors:** Yao Meng, Zhen Yang, Yaodong Quan, Shuxin Zhao, Leying Zhang, Ling Yang

**Affiliations:** School of Life Sciences and Food Engineering, Hebei University of Engineering, Handan 056038, China; mynevergiveup@126.com (Y.M.); 15031789468@163.com (Z.Y.); quanyaodong7826@163.com (Y.Q.); shuxinzhao129@gmail.com (S.Z.); zhangly056000@126.com (L.Z.)

**Keywords:** IkappaB, pregnancy, sheep, thymus

## Abstract

**Simple Summary:**

The thymus is implicated in central immune tolerance and plays essential roles during normal pregnancy. The inhibitor of the NF-κB (IκB) family is a key component of the NF-κB pathway; however, the modulation of the κB family in the maternal thymus by early gestation has not been fully understood. It has been found that there changes in the expression of the IκB family in the maternal thymus take place during early pregnancy, suggesting that IκB proteins are involved in modulating immunologic tolerance and pregnancy establishment.

**Abstract:**

The thymus is an essential component of maternal immune systems that play key roles in recognizing the placenta as immunologically foreign. The inhibitor of the NF-κB (IκB) family has essential effects on the NF-κB pathway; however, it is unclear whether early pregnancy modulates the expression of the IκB family in the thymus. In this study, maternal thymuses were sampled on day 16 of nonpregnancy and different gestation stages in the ovine, and the expression of IκB proteins was analyzed. The data showed that B cell leukemia-3 and IκBβ increased; however, IκBα, IκBε, and IKKγ deceased during gestation. Furthermore, there was an increase in IκBNS and IκBζ expression values on day 13 of pregnancy; however, this decreased on day 25 of gestation. In summary, the expression of the IκB family was modulated in the thymus during early gestation, suggesting that the maternal thymus can be associated with maternal immunologic tolerance and pregnancy establishment in ewes.

## 1. Introduction

Successful implantation and healthy pregnancy are associated with the local immune recognition of the trophoblast during gestation; however, functions of the maternal immune system that play a role in reproductive success remain controversial in humans [1]. It is through B cell tolerance against fetal antigens and placental cells that females do not identify the placenta as immune heterogeneous in mice and humans [2]. Domestic ruminant conceptus secretes interferon-tau (IFNT) to modulate the gene expression of the innate immune system, and progesterone can also be associated with a changing maternal immune function during pregnancy [3]. IFNT prevents conceptus rejection by the female by regulating the maternal innate immune system in ruminants [4]. Gene and protein expression levels of interferon-stimulated genes (ISGs), the progesterone receptor, and progesterone-induced blocking factor in the maternal immune organs were modulated by IFNT and progesterone during early pregnancy in the ovine [5].

The activation of nuclear factor kappa B (NF-κB) is associated with miscellaneous stimuli and stimulus-specific NF-κB dynamics can be related to inflammatory gene activation in macrophages [6]. NF-κB modulates maternal T-cell functions during normal pregnancy, and the dysregulation of NF-κB in the T-cell can be related to intrauterine growth restriction [7]. There is a modulation of NF-κB components in the maternal immune organs that play essential roles in ovine maternal immunoregulation and pregnancy maintenance [5]. Inhibitors of NF-κB (IκB) and IκB kinase (IKK) are the critical terminal components of NF-κB signaling, and IκB proteins include B cell leukemia-3 (BCL-3), IκBα (NFKBIA), IκBβ (NFKBIB), IκBε (NFKBIE), IKKγ (IKBKG), IκBNS (NFKBID) and IκBζ (NFKBIZ) [8]. There is a downregulation of IκBs in peripheral blood mononuclear cells (PBMCs) during normal pregnancy compared to nonpregnant females, and IκB values are downregulated more in pregnant females with preeclampsia [9]. The plasma IκBα level is downregulated in preeclamptic females compared with normotensive pregnant females; however, there is no significant difference in the plasma IκBα level between human immunodeficiency virus (HIV) positive and HIV-negative pregnant women [10]. Our previous studies reported that the expression of IκB proteins in ovine maternal liver, spleen, and lymph nodes was modulated during early pregnancy, which is associated with ovine maternal immune tolerance [5,11].

The thymus is necessary for the production of lymphocytes that differentiate into various T-cell subsets to ensure normal immune functions [12]. The thymus participates in adaptive immune responses by exporting naïve T-cells and eliminating self-reactive T-cells, which also participates in the establishment of central tolerance in humans [13]. Thymic functions vary markedly to ensure a successful pregnancy, which can be related to the development of natural regulatory T-cells and regulated by the osteoclast differentiation receptor and female sex hormones in pregnant female mice [14]. Maternal regulatory T-cells derived from the thymus circulate in mothers and promote maternal tolerance to the conceptus during gestation in mice [15]. It has been reported that expression levels of T helper cytokines, prostaglandin synthases, prolactin, and its receptor are modulated in the thymus during early gestation in sheep [16]. On the other hand, the expression of Toll-like receptor signaling members, complement components, and nucleotide-binding oligomerization domain receptors are modulated in the thymus in early gestation [17,18].

It was assumed that the expression of the IκB family was modulated in the maternal thymus during early gestation. The objective of this study was to analyze the expression of IκB proteins in the maternal thymus during early gestation in ewes, and this result could be beneficial for elucidating the thymic immunomodulation during early gestation.

## 2. Materials and Methods

### 2.1. Animal Tissue Collection

The study was carried out using Small-tail Han ewes of the same age (18 months) from September to December, and ewes were kept indoors with free access to feed and water. The experimental design was described previously [5]. Thymuses were collected after the females were euthanatized at days 13, 16, and 25 post-estrus for the pregnant ewes and on day 16 post-estrus for the nonpregnant ewes, as described previously [11]. The euthanasia of the ewes was performed by an experienced person to cut both the carotid arteries and jugular veins to bleed out the animals after electrical stunning.

### 2.2. RNA Extraction and qRT-PCR Assay

Total RNA extraction and measurements of the concentration of total RNA were performed as described previously [5], and cDNA synthesis and specific primers synthesis were carried out as described previously [16]. Amplification was carried out as described previously [5]. The specific primers were synthesized by Shanghai Sangon Biotech Co., Ltd., Shanghai, China (Table S1). The amplification efficiencies of the primer sequences were evaluated before quantification and were in an acceptable range (between 0.9 and 1.0) (please find Figure S1 Amplification curve). The 2^−ΔΔCt^ analysis method [19] was used to analyze the expression values as described previously [5].

### 2.3. Western Blot

The thymic samples were prepared on ice as described previously [16], and proteins were separated and electroblotted onto PVDF membranes (Millipore, Bedford, MA, USA). Western blot was performed, and immunospecific bands were analyzed as described previously [16] to analyze the expression of IκB proteins in the maternal thymus.

### 2.4. Immunohistochemistry Analysis

The fixed thymic tissues were prepared as described previously [16]. Immunohistochemistry analysis for IκBβ and IKKγ in the thymic tissue and stain of hematoxylin and eosin (HE) was performed as described previously [5] to detect the locations of the IκBβ and IKKγ proteins in the maternal thymus.

### 2.5. Statistical Analysis

Data for expression levels of IκB proteins were from a population with a normal distribution and were analyzed with a MIXED procedure in SAS (Version 9.1; SAS Institute, Cary, NC, USA). The Duncan method was used to test statistical significance between the groups. A *p*-value of <0.05 was considered significantly different.

## 3. Results

### 3.1. Expression of IκB Genes in the Thymus

**Figure 1:**
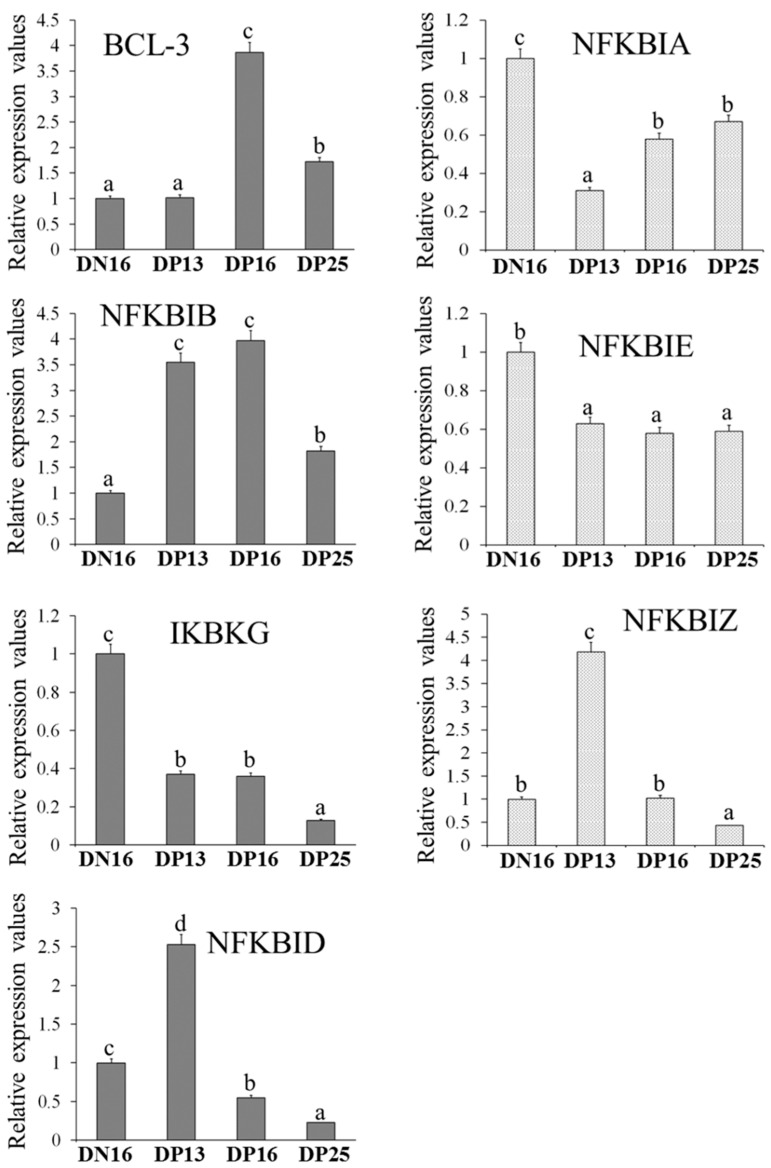
Relative expression values of IκB genes in ovine thymus. Note: Significant differences (*p* < 0.05) are indicated by different letters (a, b, c, or d) within the same color column.

IκB gene expression levels were analyzed by the qRT-PCR assay. Figure 1 (and Table S2 Relative expression values of mRNA) show that there was an increase in *BCL-3* mRNA at days 16 and 25 of pregnancy (DP16 and DP25) with a peak at DP16, and the *NFKBIB* level increased during early gestation with the highest value at DP16 (*p* < 0.05). However, there was a downregulation of *NFKBIA*, *NFKBIE*, and *IKBKG* mRNA during early gestation (*p* < 0.05). In addition, *NFKBIZ* and *NFKBID* mRNA values peaked at day 13 of the pregnancy (DP13). However, the levels at DP25 were the lowest among the four groups.

### 3.2. Expression of IκB Proteins in the Thymus

**Figure 2:**
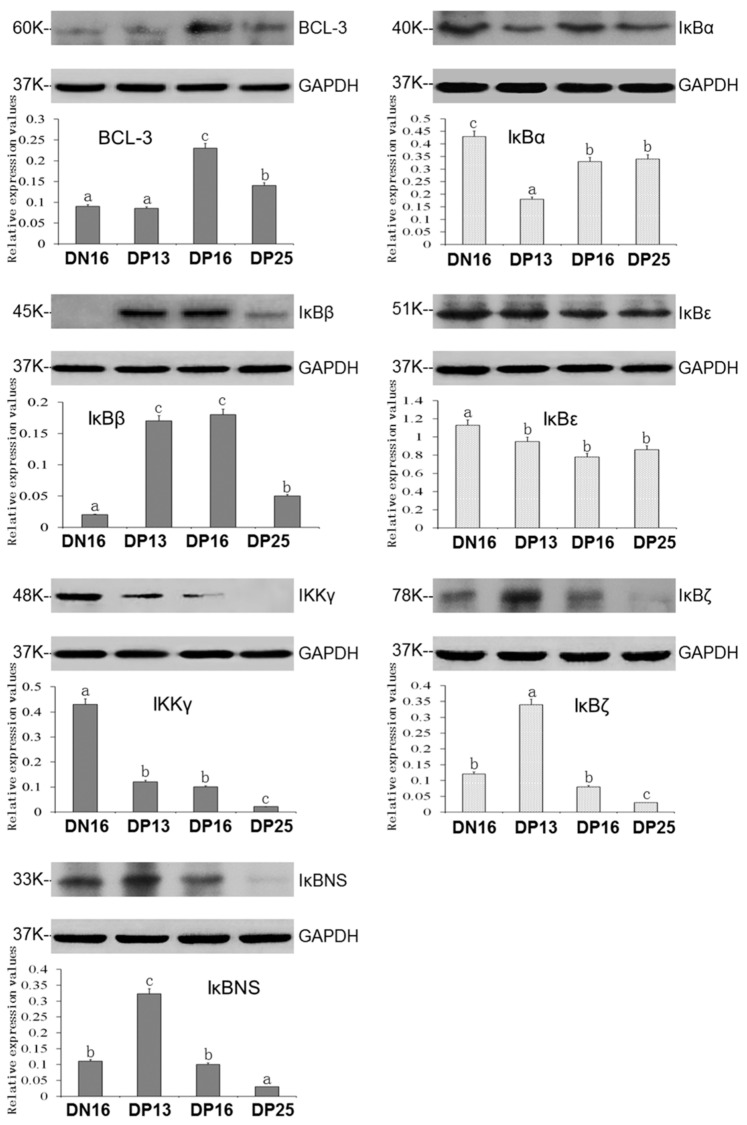
Expression of IκB proteins in the ovine thymus. Note: Significant differences (*p* < 0.05) are indicated by different superscript letters (a, b, or c) within the same color column (please find the WB full membrane in Figure S2).

IκB protein expression values were analyzed by Western blot. There were the same expression patterns of mRNA and protein levels, but there was only a slight difference between the expression of the IκBNS protein and mRNA at DN16 compared with DP16. It was revealed in Figure 2 that pregnancy stimulated the expression of the BCL-3 protein on DP16 and DP25, and the IκBβ protein was upregulated during early gestation; however, the BCL-3 and IκBβ proteins were decreased at DP25 (*p* < 0.05). However, early pregnancy suppressed the expression of IκBα, IκBε, and IKKγ proteins, and the IKKγ protein was almost undetected at DP25 (*p* < 0.05). Moreover, IκBζ and IκBNS protein values peaked at DP13 (*p* < 0.05) but were not detected at DP25.

### 3.3. Immunohistochemistry for IκBβ and IKKγ Proteins

**Figure 3:**
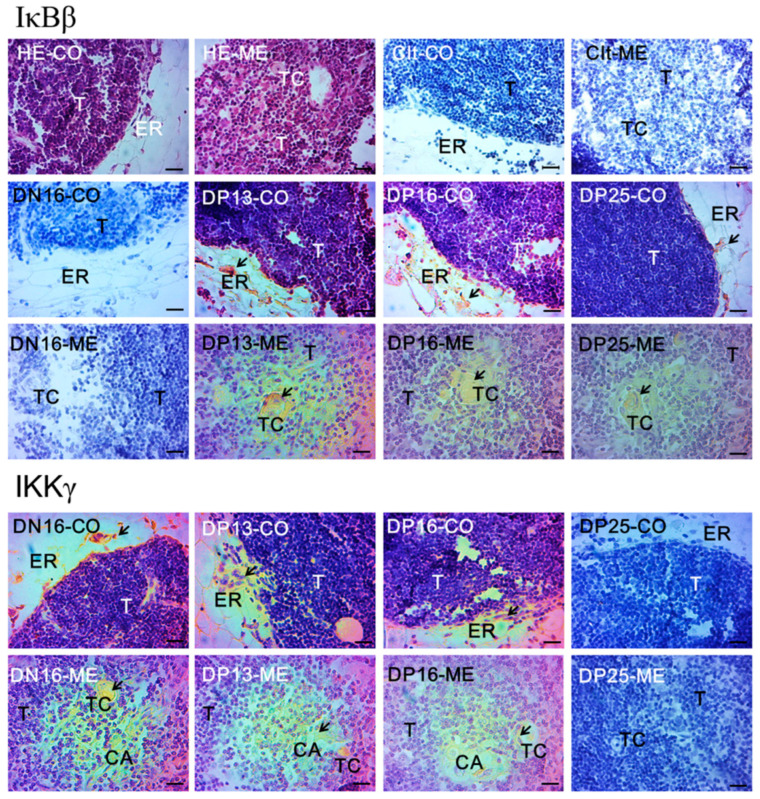
Representative immunohistochemical localization of IκBβ and IKKγ proteins in the maternal thymus. The thymus consists of the cortex (CO) and medulla (ME), and arrows indicate positive signals. Note: Bar = 20 µm; HE = stained by haematoxylin and eosin; Ctl = negative control; T = thymocyte; ER = epithelial reticular cell; CA = capillary; TC = thymic corpuscle.

Locations of IκBβ and IKKγ proteins in the thymus were analyzed by immunohistochemistry. IκBβ and IKKγ proteins were located in the epithelial reticular cells, capillaries, and thymic corpuscles. For the negative control, thymuses from day 16 of the estrous cycle (DN16) and thymuses from DP13, DP16, and DP25 were observed with the staining intensities for the IκBβ protein at 0, 0, 2, 2, and 1, and those for the IKKγ protein at 0, 3, 1, 1 and 0, respectively (Figure 3). The staining intensities of 0, 1, 2, and 3 were negative, weak, strong, and stronger, respectively.

## 4. Discussion

It is through binding to NF-κB, p50, and p52 homodimers that BCL-3 is involved in regulating the atypical NF-κB pathway, as well as the transcriptional inhibition or activation of NF-κB target genes [20]. BCL-3 inhibits regulatory T-cell accumulation and differentiation, which are implicated in immune tolerance in mice, and can be used for immunotherapy [21]. BCL-3 plays key roles in the Th1-type adaptive immune response and immune system development, which are essential for the development of autoimmune and inflammatory diseases [22]. BCL-3 is a negative nuclear regulator of tumor necrosis factor (TNF)-α and is expressed in the murine uterus at diestrus, which is required for the preparation of pregnancy [23]. BCL-3 regulates the expression of TNF-α to inhibit the innate immune response; however, the overexpression of BCL-3 is associated with early-onset preeclampsia [24]. BCL-3 promotes central tolerance and is an accommodator of immunologic tolerance in the periphery [25]. Early pregnancy modulates BCL-3 in the maternal liver, which regulates the maternal hepatic function to prepare for pregnancy establishment in the ovine [5]. BCL-3 expression increases in the maternal spleen but decreases in the maternal lymph nodes during the early gestation of ewes, suggesting that BCL-3 is implicated in the splenic adaptive immunity and immune tolerance of lymph nodes [11]. BCL-3 is expressed in thymocytes, which is associated with the DNA binding activity of NF-κB1 homodimers which affects the biological activities of NF-κB1 [26]. Furthermore, BCL-3 participates in thymus development and organization and is implicated in innate and adoptive immunoregulation [27]. In this study, BCL-3 expression increased during early gestation. However, there was a decline at DP25. Thus, the increase in BCL-3 could be related to maternal immunologic tolerance; however, its decline at DP25 may contribute to modulating maternal adaptive immunity during early pregnancy.

The phosphorylation of IκBα results in μ-calpain-mediated IκBα degradation, which activates the NF-κB-dependent transcription of PD-L1 in human glioblastoma cells and increases tumor immune evasion [28]. Regulated in development and DNA damage responses 1, interacts with IκBα to induce atypical NF-κB activation, aggravating endotoxin-induced inflammation [29]. Mutations of IκBα can cause ectodermal dysplasia with immunodeficiency, suggesting that IκBα plays a critical role in reconstituting the immune function [30]. IκBα in glioblastoma cells promotes CD8^+^ T cell activation and regulates tumor immune evasion [28]. The level of IκBα in PBMCs from pregnant women was downregulated compared with nonpregnant controls; however, the IκBα level decreased more in preeclampsia [10]. Low molecular weight seleno-aminopolysaccharides can significantly enhance thymus indices and promote IκBα phosphorylation to exert immunomodulatory activity on immunosuppressive mice [31]. IκBα was elevated in preeclamptic placentas compared with the control group [32]. The IκBα level was higher in PBMCs from nonpregnant women than from pregnant females [9]. During ovine early gestation, IκBα expression was upregulated in the maternal spleen and increased but then decreased in the maternal lymph node, which could be associated with splenic B-cell maturation and maternal immunoregulation [11]. Early pregnancy stimulates IκBα expression in the ovine maternal liver, which is beneficial for pregnancy establishment [5]. Our data showed that early gestation suppressed IκBα expression. Thus, the downregulation of IκBα can be associated with maternal immunomodulation and pregnancy establishment.

IκBβ improves the production of the low-affinity NF-κB/RelA homodimer, which regulates inflammatory and immune responses of NF-κB signaling [33]. The overexpression of IκBβ attenuates lipopolysaccharide-stimulated IκBβ-NFκB signaling in mice [34]. IκBβ is implicated in precluding oxidant stress-stimulated cell death by blocking NF-κB signaling [35]. The IκBβ level was decreased in PBMCs from the pregnant females with preeclampsia, suggesting that IκBβ can be related to the fetus avoiding maternal rejection throughout pregnancy [9]. A penetrating brain injury induces the expression of IκBβ, which can be associated with a decrease in estrogen and can increase cell survival levels [36]. In general, during early pregnancy, the serum estrogen concentration is low, but the progesterone concentration is high in ruminants. However, it has been reported that the IκBβ expression level peaks on DP16 in the maternal liver and lymph nodes during the early gestation of ewes, which are associated with the IFNT from conceptus, and are important for ovine pregnancy recognition [5,11]. The data showed that the IκBβ expression level was upregulated during early gestation, and the IκBβ protein was located in epithelial reticular cells, capillaries, and thymic corpuscles. Thus, the increase in IκBβ was associated with the downregulation of NF-κB signaling, which could be related to the low concentration of serum estrogen and high concentration of progesterone, which is favorable for the fetus to avoid maternal immune rejection.

IκBε restricts c-Rel proteins in the cytoplasm, sequesters NF-κB p65 homodimers, and functions in the nucleus to terminate NF-κB-dependent transcriptional activation, controlling the biological activity of NF-κB signaling [37]. IκBε is implicated in modulating B-cell development and function, and NFKBIE deficiency results in NF-κB activation in mice [38]. IκBε exists as negative feedback on NF-κB signaling and can dampen IκBα-mediated impacts on NF-κB activity in response to the transient stimulation in experimental and computational models [39]. The IκBε protein shuttles between the nucleus and cytoplasm to control the nucleocytoplasmic distribution of NF-κB/Rel proteins [40]. TNF-α and the insulin-like growth factor-I change NFKBIE DNA methylation in the human placental cell line and also lead to pathological alterations in the placenta, which results in preeclampsia [41]. An IκBε-deficiency leads to the higher expression of c-Rel by mouse T- and B-cells, which impacts strongly on B- and T-cell development [42]. The IκBε level peaks at DP16 in the maternal liver and lymph nodes, which is involved in hepatic homeostasis and regeneration, as well as the immunoregulation of the maternal liver and lymph nodes. However, the IκBε level upregulates in the maternal spleen, which is associated with pregnancy establishment during ovine early gestation [5,11]. Our data showed that IκBε was downregulated in the maternal thymus during early gestation. Thus, the downregulation of IκBε was implicated in the regulation of maternal thymic T-cell development and was related to maternal immunomodulation and pregnancy recognition.

NEMO plays a key role in modulating NF-κB-mediated signaling through the transmission of extracellular or intracellular signals and the regulation of IKK complex activity [43]. NEMO regulates IκB kinase activation to phosphorylate NF-κB inhibitors IκBs that modulate NF-κB signaling to participate in immune and inflammatory responses [44]. The downregulation of NEMO suppresses NF-κB signal transduction to modulate inflammatory and immune responses [45]. NEMO (also known as IKKγ) deficiency results in an increase in the ISG15 expression, which is a type I interferon signature [46]. There is an increase in the ISG15 protein in the maternal thymus, which is induced by IFNT secreted from the conceptus during early pregnancy [5]. The upregulation of the *NEMO* gene in the maternal blood of women with preeclampsia suggests that NEMO can be associated with preeclampsia development [47]. It is through suppressing the NEMO/NF-κB pathway that peptidyl arginine deiminase 4 knockdown inhibits inflammation in trophoblast cells to reduce preeclampsia development in vitro [48]. The NF-κB-essential-modulator (NEMO) is involved in the regulation of thymocyte selection by the cylidromatosis tumor suppressor [49]. The IKKγ protein peaks at DP16 in the ovine maternal liver and lymph nodes, which are involved in the regulation of inflammatory responses to the liver and lymph nodes. However, the IKKγ level gradually increases in the maternal spleen during early pregnancy, which can be related to the maternal splenic immunoregulation for pregnancy establishment [5,11]. Our results showed that IKKγ expression was suppressed in early pregnancy, and the IKKγ protein was located in epithelial reticular cells, capillaries, and thymic corpuscles. Thus, a lower level of IKKγ could be related to the pregnancy recognition signal (IFNT), and the downregulation of IKKγ was helpful for pregnancy establishment and maintenance.

IκBζ, encoded by NFKBIZ, can modulate Th17 development in T-cells while also participating in the activation of natural killer cells [50]. The upregulation of the transcription factor, NFKBIZ, was found to enhance immunoglobulin G1 (IgG1) production in Chinese hamster ovary cell lines, while IgG1 mediated antibody responses and had effects on the innate immune system [51]. IκBζ positively or negatively modulated the transcriptional activity of NF-κB subunits in a gene-specific manner and was involved in activating the acquired immune system [52]. IκBζ can regulate IFN-γ expression in T-cells and participates in regulating the function of Treg cells [53]. There is a high IκBζ expression in the inflammation site, and IκBζ is involved in CD4^+^ T cell differentiation [54]. The upregulation of the Th1 cytokine in PBMCs from aborted females indicated that the Th1 cytokine was adverse to normal pregnancy [55]. Early pregnancy suppresses Th1 immunity (IFN-γ) in the PBMCs, which is associated with IFNT and progesterone in cattle [56]. The IκBζ protein expression is enhanced in the maternal liver on DP13 and DP16 but declines on DP25, which can be related to IFNT and hepatic inflammatory response in sheep [5]. IκBζ expression increases in the spleen and lymph nodes during early gestation, which can be associated with the negative modulation of NF-κB activity, which contributes to maternal immune tolerance in ewes [11]. The data showed that the IκBζ level was upregulated on DP13 but downregulated on DP25 in the maternal thymus. Thus, the increase in thymic IκBζ was related to maternal immunoregulation but decreased for IκBζ at DP25, which was favorable for pregnancy establishment.

IκBNS is essential for the selection and survival of immature thymocytes and participates in the regulation of Treg cell development and NF-κB activity [57]. IκBNS loss enhances mitochondrial metabolism and downregulates autophagic capacity in B-cells, which can have negative effects on innate-like B-cell development [58]. IκBNS can remodel the uterus for blastocyst implantation under the transcriptional control of NF-κB via negatively regulating the transcription of IL-6 in mice [21,22]. IκBNS and c-Rel are required in governing thymic Treg cell development through the NF-κB route and forkhead box P3 (Foxp 3) and CD25 route [59]. The allelic variant of IκBNS results in the acceleration of type 1 diabetes onset and autoreactive CD8 T-cell deletion in the mouse thymus, which can be related to a decrease in the peripheral Tregs frequency [60]. During early gestation, IκBNS expression levels are upregulated on DP13 but decline on DP25 in the ovine liver, spleen, and lymph nodes, which are involved in regulating the functions of the maternal liver, spleen, and lymph nodes, as well as peripheral tolerance [5,11]. Our results showed that IκBNS increased at DP13 but was downregulated from DP16 to DP25. Thus, the increase in IκBNS at day 13 of pregnancy could be related to the regulation of thymic Treg cell development; however, the decrease at DP16 and DP25 was involved in maternal thymic immunoregulation.

**Figure 4:**
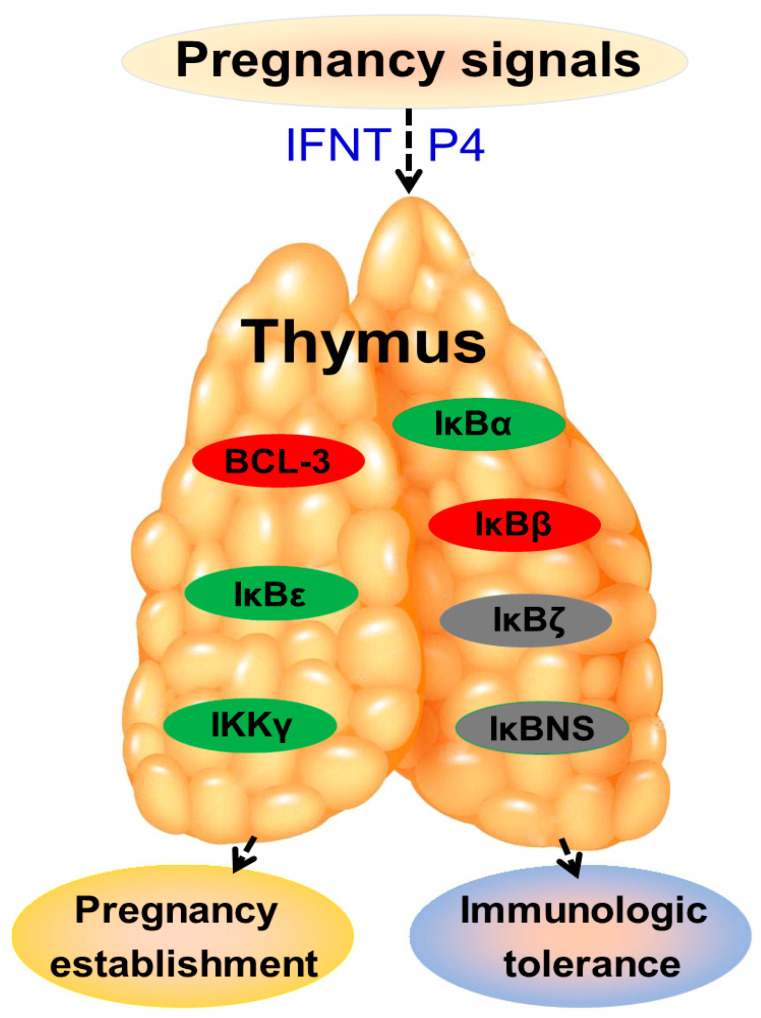
Sketch of IkappaB proteins in the maternal thymus during early gestation in sheep. Early pregnancy signals, including interferon-tau (IFNT) and progesterone (P4), induce the changed expression of IkappaB (IκB) proteins through blood circulation, which is related to maternal immunologic tolerance and pregnancy establishment. Red, stimulators; Green, negative regulators; Gray, upregulated and then downregulated.

Type I interferons induce ISG expression to establish and regulate host defense against microbial infection and are necessary for modulating chromatin structure and function [61]. As a type I interferon, IFNT has effects on the ovine maternal thymus to stimulate ISG15 expression, and progesterone affects thymic immune functions by modulating the progesterone-induced blocking factor expression via the progesterone receptor [62]. Early pregnancy affects the expression of the NF-κB family and improves NF-κB1, RelB, and c-Rel expression but suppresses NF-κB2 and RelA expression in the thymus of ewes [63]. NF-κB is important for thymic T-cell maturation and proliferation, and type I IFN can improve T-cells’ response to inflammatory factors in the thymic medulla [64]. Early pregnancy signals, including IFNT and progesterone (Figure 4), regulate maternal thymic immune function through blood circulation, which upregulates BCL-3 and IκBβ levels, downregulates IκBα, IκBε and IKKγ levels, and upregulates and then downregulates IκBζ and IκBNS levels in the maternal thymus. Furthermore, these changes regulate maternal thymic Treg cell development to result in maternal thymic immune tolerance and participate in maternal central tolerance and pregnancy maintenance.

## 5. Conclusions

During early gestation, BCL-3 and IκBβ were upregulated; however, the expression of IκBα, IκBε, and IKKγ was downregulated, and the expression of IκBζ and IκBNS increased and then decreased in the maternal thymus. Therefore, early pregnancy influenced the expression of the IκB family, which can be associated with the regulation of maternal thymic Treg cell development and immune tolerance, which is helpful for pregnancy recognition and establishment.

## Data Availability

Not applicable.

## Supplementary Materials

The following supporting information can be downloaded at: https://www.mdpi.com/article/10.3390/vetsci10070462/s1, Figure S1: Amplification curve; Figure S2: Original Western Blot Figure for Figure 2; Table S1: Primers used for RT-qPCR; Table S2: Relative expression values of mRNA.

## References

[1] Moffett A., Shreeve N. (2022). Local immune recognition of trophoblast in early human pregnancy: Controversies and questions. Nat. Rev. Immunol..

[2] Rizzuto G., Brooks J.F., Tuomivaara S.T., McIntyre T.I., Ma S., Rideaux D., Zikherman J., Fisher S.J., Erlebacher A. (2022). Establishment of fetomaternal tolerance through glycan-mediated B cell suppression. Nature.

[3] Ott T.L. (2020). Immunological detection of pregnancy: Evidence for systemic immune modulation during early pregnancy in ruminants. Theriogenology.

[4] Rocha C.C., da Silveira J.C., Forde N., Binelli M., Pugliesi G. (2021). Conceptus-modulated innate immune function during early pregnancy in ruminants: A review. Anim. Reprod..

[5] Cai C., Ren Y., Cao J., Fang S., Zhang L., Yang L. (2023). Expression of IkappaB family in the ovine liver during early pregnancy. Animals.

[6] Cheng Q.J., Ohta S., Sheu K.M., Spreafico R., Adelaja A., Taylor B., Hoffmann A. (2021). NF-κB dynamics determine the stimulus specificity of epigenomic reprogramming in macrophages. Science.

[7] Ariyakumar G., Morris J.M., McKelvey K.J., Ashton A.W., McCracken S.A. (2021). NF-κB regulation in maternal immunity during normal and IUGR pregnancies. Sci. Rep..

[8] Mulero M.C., Huxford T., Ghosh G. (2019). NF-κB, IκB, and IKK: Integral components of immune system signaling. Adv. Exp. Med. Biol..

[9] McCracken S.A., Drury C.L., Lee H.S., Morris J.M. (2003). Pregnancy is associated with suppression of the nuclear factor kappaB/IkappaB activation pathway in peripheral blood mononuclear cells. J. Reprod. Immunol..

[10] Zozo B., Govender N., Moodley J., Naicker T. (2021). Expression of plasma nuclear factor-kappa B cells (NF-κB) and Inhibitory subunit kappa B alpha (IκB-α) in HIV-associated pre-eclampsia. Hypertens Pregnancy.

[11] Fang S., Cai C., Bai Y., Zhang L., Yang L. (2023). Early Pregnancy regulates expression of IkappaB family in ovine spleen and lymph nodes. Int. J. Mol. Sci..

[12] Miller J.F.A.P. (2020). The function of the thymus and its impact on modern medicine. Science.

[13] Thapa P., Farber D.L. (2019). The role of the thymus in the immune response. Thorac. Surg. Clin..

[14] Paolino M., Koglgruber R., Cronin S.J.F., Uribesalgo I., Rauscher E., Harreiter J., Schuster M., Bancher-Todesca D., Pranjic B., Novatchkova M. (2021). RANK links thymic regulatory T cells to fetal loss and gestational diabetes in pregnancy. Nature.

[15] Ahn S.H., Nguyen S.L., Petroff M.G. (2020). Exploring the origin and antigenic specificity of maternal regulatory T cells in pregnancy. Front. Immunol..

[16] Feng P., Wu J., Ren Y., Zhang L., Cao J., Yang L. (2022). Early pregnancy regulates the expression of prolactin and its receptor in the thymus, the liver, the spleen and lymph nodes in sheep. Domest. Anim. Endocrinol..

[17] Zhang L., Li Y., Zhao Z., Cai J., Zhao S., Yang L. (2022). Modulation of nod-like receptor expression in the thymus during early pregnancy in ewes. Vaccines.

[18] Zhang L., Zhang Q., Wang H., Feng P., Yang G., Yang L. (2022). Effects of early pregnancy on the complement system in the ovine thymus. Vet. Res. Commun..

[19] Livak K.J., Schmittgen T.D. (2001). Analysis of relative gene expression data using real-time quantitative PCR and the 2(-Delta Delta C(T)) method. Methods..

[20] Liu H., Zeng L., Yang Y., Guo C., Wang H. (2022). Bcl-3: A double-edged sword in immune cells and inflammation. Front. Immunol..

[21] Tang W., Saret S., Tian R., Wang H., Claudio E., Murphy P.M., Siebenlist U. (2021). Bcl-3 suppresses differentiation of RORγt+ regulatory T cells. Immunol. Cell Biol..

[22] Tassi I., Claudio E., Wang H., Tang W., Ha H.L., Saret S., Sher A., Jankovic D., Siebenlist U. (2015). Adaptive immune-mediated host resistance to Toxoplasma gondii is governed by the NF-κB regulator Bcl-3 in dendritic cells. Eur. J. Immunol..

[23] Sierra-Mondragón E., Gómez-Chávez F., Murrieta-Coxca M., Vázquez-Sánchez E.A., Martínez-Torres I., Cancino-Díaz M.E., Rojas-Espinosa O., Cancino-Díaz J.C., Reyes-Sánchez J.L., Rodríguez-Muñóz R. (2015). Low expression of IL-6 and TNF-α correlates with the presence of the nuclear regulators of NF-κB, IκBNS and BCL-3, in the uterus of mice. Mol. Immunol..

[24] Gómez-Chávez F., Correa D., Navarrete-Meneses P., Cancino-Diaz J.C., Cancino-Diaz M.E., Rodríguez-Martínez S. (2021). NF-κB and its regulators during pregnancy. Front. Immunol..

[25] Zhang X., Wang H., Claudio E., Brown K., Siebenlist U. (2007). A role for the IkappaB family member Bcl-3 in the control of central immunologic tolerance. Immunity.

[26] Caamaño J.H., Perez P., Lira S.A., Bravo R. (1996). Constitutive expression of Bc1-3 in thymocytes increases the DNA binding of NF-kappaB1 (p50) homodimers in vivo. Mol. Cell. Biol..

[27] Paxian S., Merkle H., Riemann M., Wilda M., Adler G., Hameister H., Liptay S., Pfeffer K., Schmid R.M. (2002). Abnormal organogenesis of Peyer’s patches in mice deficient for NF-kappaB1, NF-kappaB2, and Bcl-3. Gastroenterology.

[28] Guo D., Tong Y., Jiang X., Meng Y., Jiang H., Du L., Wu Q., Li S., Luo S., Li M. (2022). Aerobic glycolysis promotes tumor immune evasion by hexokinase2-mediated phosphorylation of IκBα. Cell Metab..

[29] Lee D.K., Kim J.H., Kim J., Choi S., Park M., Park W., Kim S., Lee K.S., Kim T., Jung J. (2018). REDD-1 aggravates endotoxin-induced inflammation via atypical NF-κB activation. FASEB J..

[30] Mooster J.L., Le Bras S., Massaad M.J., Jabara H., Yoon J., Galand C., Heesters B.A., Burton O.T., Mattoo H., Manis J. (2015). Defective lymphoid organogenesis underlies the immune deficiency caused by a heterozygous S32I mutation in IκBα. J. Exp. Med..

[31] Wen Z.S., Tang Z., Gu L.X., Xiang X.W., Qu Y.L. (2019). Immunomodulatory effect of low molecular-weight seleno-aminopolysaccharide on immunosuppressive mice. Int. J. Biol. Macromol..

[32] Sakowicz A., Bralewska M., Pietrucha T., Habrowska-Górczyńska D.E., Piastowska-Ciesielska A.W., Gach A., Rybak-Krzyszkowska M., Witas P.J., Huras H., Grzesiak M. (2020). Canonical, Non-canonical and atypical pathways of nuclear factor кb activation in preeclampsia. Int. J. Mol. Sci..

[33] Tsui R., Kearns J.D., Lynch C., Vu D., Ngo K.A., Basak S., Ghosh G., Hoffmann A. (2015). IκBβ enhances the generation of the low-affinity NFκB/RelA homodimer. Nat. Commun..

[34] McKenna S., Wright C.J. (2015). Inhibiting IκBβ-NFκB signaling attenuates the expression of select pro-inflammatory genes. J. Cell Sci..

[35] Wright C.J., Agboke F., Muthu M., Michaelis K.A., Mundy M.A., La P., Yang G., Dennery P.A. (2012). Nuclear factor-κB (NF-κB) inhibitory protein IκBβ determines apoptotic cell death following exposure to oxidative stress. J. Biol. Chem..

[36] Cook S., Hung V., Duncan K.A. (2018). Crosstalk between estrogen withdrawal and NFκB signaling following penetrating brain injury. Neuroimmunomodulation.

[37] Simeonidis S., Liang S., Chen G., Thanos D. (1997). Cloning and functional characterization of mouse IkappaBepsilon. Proc. Natl. Acad. Sci. USA.

[38] Della-Valle V., Roos-Weil D., Scourzic L., Mouly E., Aid Z., Darwiche W., Lecluse Y., Damm F., Mémet S., Mercher T. (2020). Nfkbie-deficiency leads to increased susceptibility to develop B-cell lymphoproliferative disorders in aged mice. Blood Cancer J..

[39] Kearns J.D., Basak S., Werner S.L., Huang C.S., Hoffmann A. (2006). IkappaBepsilon provides negative feedback to control NF-kappaB oscillations, signaling dynamics, and inflammatory gene expression. J. Cell. Biol..

[40] Lee S.H., Hannink M. (2002). Characterization of the nuclear import and export functions of Ikappa B(epsilon). J. Biol. Chem..

[41] Tanaka K., Nakabayashi K., Kawai T., Tanigaki S., Matsumoto K., Hata K., Kobayashi Y. (2020). Gene expression and DNA methylation changes in BeWo cells dependent on tumor necrosis factor-α and insulin-like growth factor-I. Hum. Cell..

[42] Clark J.M., Aleksiyadis K., Martin A., McNamee K., Tharmalingam T., Williams R.O., Mémet S., Cope A.P. (2011). Inhibitor of kappa B epsilon (IκBε) is a non-redundant regulator of c-Rel-dependent gene expression in murine T and B cells. PLoS ONE.

[43] Maubach G., Schmädicke A.C., Naumann M. (2017). NEMO links nuclear factor-κB to human diseases. Trends Mol. Med..

[44] Du M., Ea C.K., Fang Y., Chen Z.J. (2022). Liquid phase separation of NEMO induced by polyubiquitin chains activates NF-κB. Mol. Cell..

[45] Wackernagel L.M., Abdi Sarabi M., Weinert S., Zuschratter W., Richter K., Fischer K.D., Braun-Dullaeus R.C., Medunjanin S. (2022). IKKγ/NEMO localization into multivesicular bodies. Int. J. Mol. Sci..

[46] Surucu Yilmaz N., Bilgic Eltan S., Kayaoglu B., Geckin B., Heredia R.J., Sefer A.P., Kiykim A., Nain E., Kasap N., Dogru O. (2022). Low density granulocytes and dysregulated neutrophils driving autoinflammatory manifestations in NEMO deficiency. J. Clin. Immunol..

[47] Sakowicz A., Hejduk P., Pietrucha T., Nowakowska M., Płuciennik E., Pospiech K., Gach A., Rybak-Krzyszkowska M., Sakowicz B., Kaminski M. (2016). Finding NEMO in preeclampsia. Am. J. Obstet. Gynecol..

[48] Zeng M., Xu M., Li X., Li J., Liu Y. (2022). PAD4 silencing inhibits inflammation whilst promoting trophoblast cell invasion and migration by inactivating the NEMO/NF-κB pathway. Exp. Ther. Med..

[49] Tsagaratou A., Grammenoudi S., Mosialos G. (2011). Differential requirement of IKK2 for CYLD-dependent representation of thymic and peripheral T-cell populations. Eur. J. Immunol..

[50] Miyake T., Satoh T., Kato H., Matsushita K., Kumagai Y., Vandenbon A., Tani T., Muta T., Akira S., Takeuchi O. (2010). IκBζ is essential for natural killer cell activation in response to IL-12 and IL-18. Proc. Natl. Acad. Sci. USA.

[51] Onitsuka M., Kinoshita Y., Nishizawa A., Tsutsui T., Omasa T. (2018). Enhanced IgG1 production by overexpression of nuclear factor kappa B inhibitor zeta (NFKBIZ) in Chinese hamster ovary cells. Cytotechnology.

[52] Muta T. (2006). IkappaB-zeta: An inducible regulator of nuclear factor-kappaB. Vitam. Horm..

[53] MaruYama T., Kobayashi S., Ogasawara K., Yoshimura A., Chen W., Muta T. (2015). Control of IFN-γ production and regulatory function by the inducible nuclear protein IκB-ζ in T cells. J. Leukoc. Biol..

[54] Ahn J.H., Cho J., Kwon B.E., Lee G.S., Yoon S.I., Kang S.G., Kim P.H., Kweon M.N., Yang H., Vallance B.A. (2019). IκBζ facilitates protective immunity against Salmonella infection via Th1 differentiation and IgG production. Sci. Rep..

[55] Raghupathy R., Makhseed M., Azizieh F., Omu A., Gupta M., Farhat R. (2000). Cytokine production by maternal lymphocytes during normal human pregnancy and in unexplained recurrent spontaneous abortion. Hum. Reprod..

[56] Yang L., Wang Y., Li S., Zhu M., He K., Yao X., Zhang L. (2018). Differential expression of interferon-gamma, IL-4 and IL-10 in peripheral blood mononuclear cells during early pregnancy of the bovine. Reprod. Biol..

[57] Sengupta S., Haczku A. (2017). Targeting IκBNS in allergic asthma: Where it resides, matters. Allergy.

[58] Erikson E., Ádori M., Khoenkhoen S., Zhang J., Rorbach J., Castro Dopico X., Karlsson Hedestam G. (2022). Impaired plasma cell differentiation associates with increased oxidative metabolism in IκBNS-deficient B cells. Cell Immunol..

[59] Schuster M., Plaza-Sirvent C., Visekruna A., Huehn J., Schmitz I. (2019). Generation of Foxp3+CD25- regulatory T-cell precursors requires c-Rel and IκBNS. Front. Immunol..

[60] Dwyer J.R., Racine J.J., Chapman H.D., Quinlan A., Presa M., Stafford G.A., Schmitz I., Serreze D.V. (2022). Nfkbid overexpression in nonobese diabetic mice elicits complete type 1 diabetes resistance in part associated with enhanced thymic deletion of pathogenic CD8 T cells and increased numbers and activity of regulatory T cells. J. Immunol..

[61] Chen K., Liu J., Cao X. (2017). Regulation of type I interferon signaling in immunity and inflammation: A comprehensive review. J. Autoimmun..

[62] Zhang L., Xue J., Wang Q., Lv W., Mi H., Liu Y., Yang L. (2018). Changes in expression of ISG15, progesterone receptor and progesterone-induced blocking factor in ovine thymus during early pregnancy. Theriogenology.

[63] Yang L., Cai C., Fang S., Hao S., Zhang T., Zhang L. (2022). Changes in expression of nuclear factor kappa B subunits in the ovine thymus during early pregnancy. Sci. Rep..

[64] Xing Y., Wang X., Jameson S.C., Hogquist K.A. (2016). Late stages of T cell maturation in the thymus involve NF-κB and tonic type I interferon signaling. Nat. Immunol..

