# Supplementary material for: Regulation of IkappaB Protein Expression by Early Gestation in the Thymus of Ewes
Source: Vet Sci. 2023 Jul 13;10(7):462. doi: 10.3390/vetsci10070462 (PMC10384501; doi:10.3390/vetsci10070462)

Figure S2 Original Western Blot Figure for Figure 2

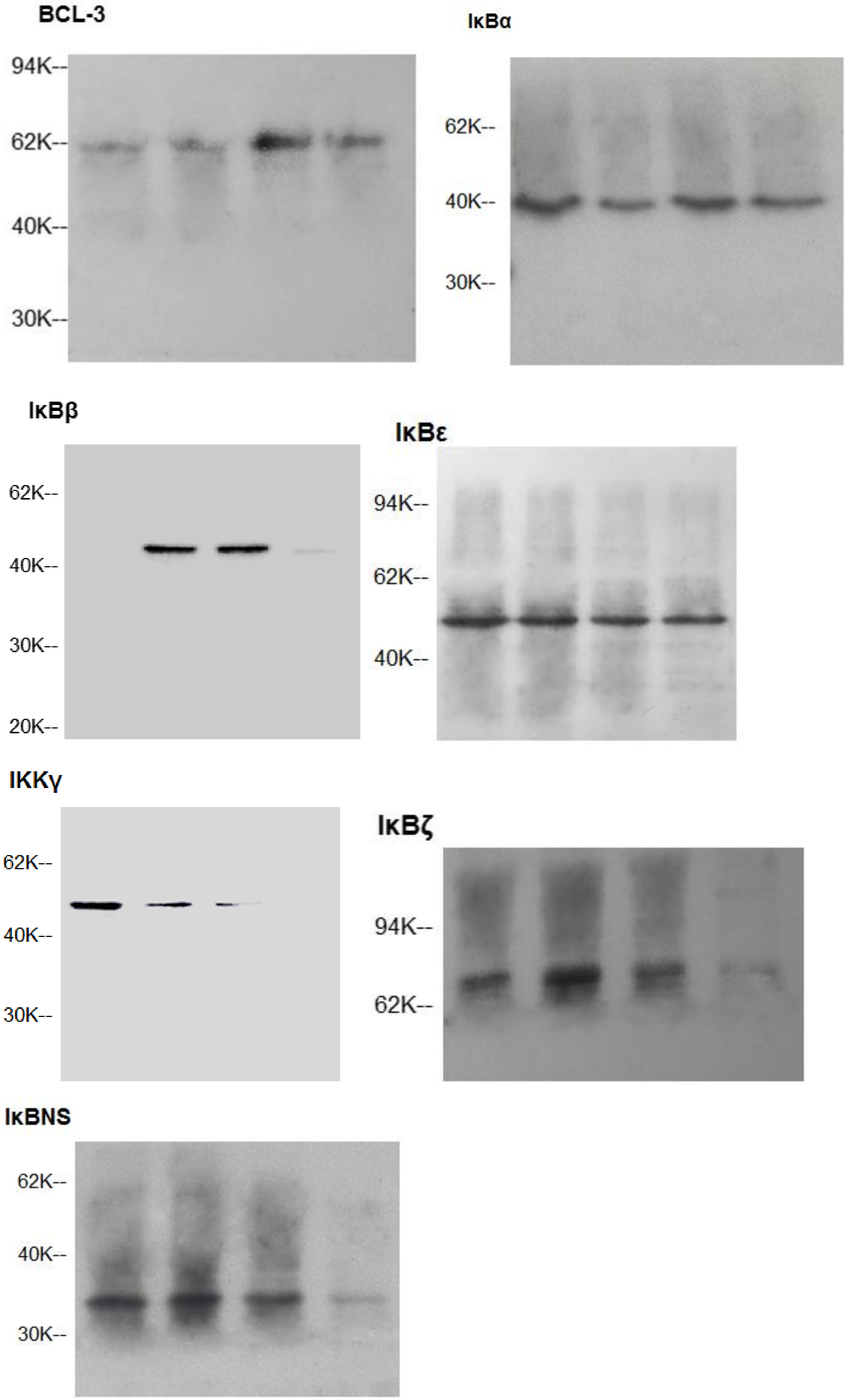

GAPDH-BCL-3

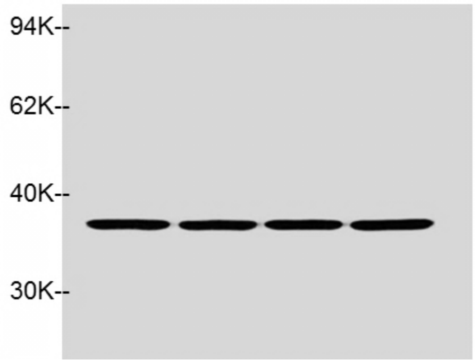

GAPDH-IκBα

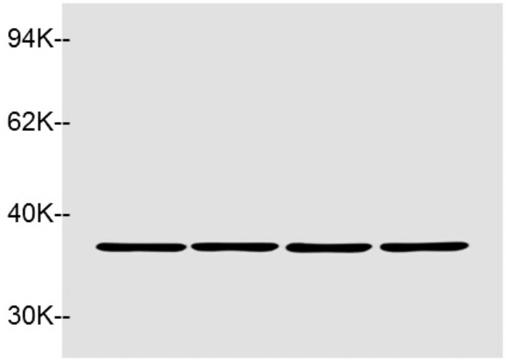

GAPDH-IκBβ

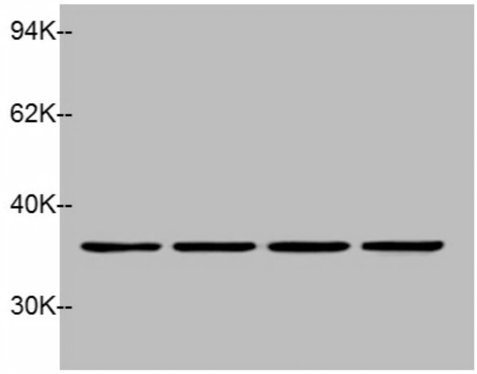

GAPDH-IκBε

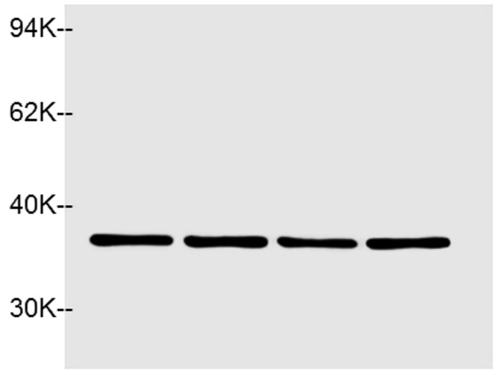

GAPDH-IKKγ

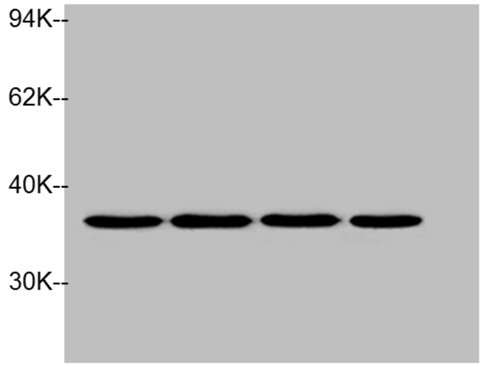

GAPDH-IκBζ

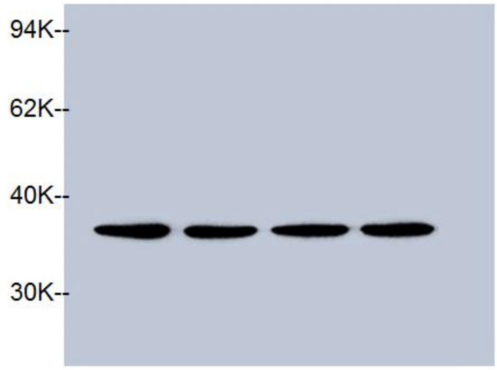

GAPDH-IκBNS

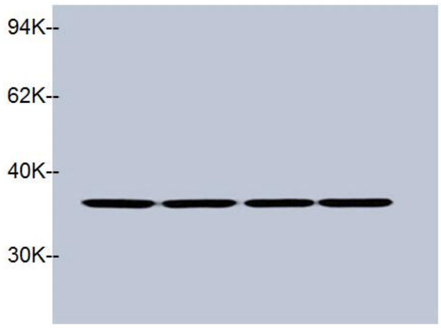

Supplement: Supplementary file 1 [file vetsci-10-00462-s001.zip › Figure S2 Original Western Blot Figure for Figure 2.pdf]
