# Supplementary material for: Regulation of IkappaB Protein Expression by Early Gestation in the Thymus of Ewes
Source: Vet Sci. 2023 Jul 13;10(7):462. doi: 10.3390/vetsci10070462 (PMC10384501; doi:10.3390/vetsci10070462)
Supplement: Supplementary file 1 [file vetsci-10-00462-s001.zip › Table S1 Primers.pdf]

Table S1 Primers used for RT-qPCR

Gene	Primer	Sequence	Size (bp)	Accession numbers
BCL-3	Forward	GCGACCAGAGGCAATTTACTACCAG	98	XM_027978453.2
	Reverse	GAGGTGTAGGCAAGTTCAGCAGAG		
NFKBIA	Forward	AGGACGAGGAGTATGAGCAGATGG	130	NM_001166184.1
	Reverse	GCCAAGTGCAGGAACGAGTCTC		
NFKBIB	Forward	CCCCAAGACCTACCTCGCTCAG	119	XM_027978262.2
	Reverse	TCCAGTCCTCTTCACTCTCATCCTC		
NFKBIE	Forward	GCACTCACGTACATTTCCGAGGAC	97	XM_042236979.1
	Reverse	GCAGCAGAGCCAGGCAATACAG		
IKBKG	Forward	GGGCAACCAGAGGGAGGAGAAG	146	XM_027963334.2
	Reverse	GGCATGTCTTCAGGCGTTCCAC		
NFKBIZ	Forward	GCAAAGGCGTACAATGGAAACACC	137	NM_001306117.1
	Reverse	GGCTGCTCGTTCTCCAAGTTCC		
NFKBID	Forward	ACATTCGTGAGCATAAGGGCAAGAC	114	XM_027977435.2
	Reverse	GATGGTCAGTGGCATTGGGTTCC		
GAPDH	Forward	GGGTCATCATCTCTGCACCT	176	NM_001190390.1
	Reverse	GGTCATAAGTCCCTCCACGA		
