# Supplementary material for: Regulation of IkappaB Protein Expression by Early Gestation in the Thymus of Ewes
Source: Vet Sci. 2023 Jul 13;10(7):462. doi: 10.3390/vetsci10070462 (PMC10384501; doi:10.3390/vetsci10070462)
Supplement: Supplementary file 1 [file vetsci-10-00462-s001.zip › Table S2 Relative expression values of mRNA.pdf]

Table S2 Relative expression values of mRNA

Item	DN16	DP13	DP16	DP25
BCL-3	1	1.02	3.87	1.72
NFKBIA	1	0.31	0.58	0.67
NFKBIB	1	3.55	3.97	1.82
NFKBIE	1	0.63	0.58	0.59
IKBKG	1	0.37	0.36	0.13
NFKBIZ	1	4.18	1.03	0.43
NFKBID	1	2.53	0.55	0.23
