# Supplementary figures and images for: Regulation of IkappaB Protein Expression by Early Gestation in the Thymus of Ewes
Source: Vet Sci. 2023 Jul 13;10(7):462. doi: 10.3390/vetsci10070462 (PMC10384501; doi:10.3390/vetsci10070462)

## Amplification curve

### 1. BCL-3

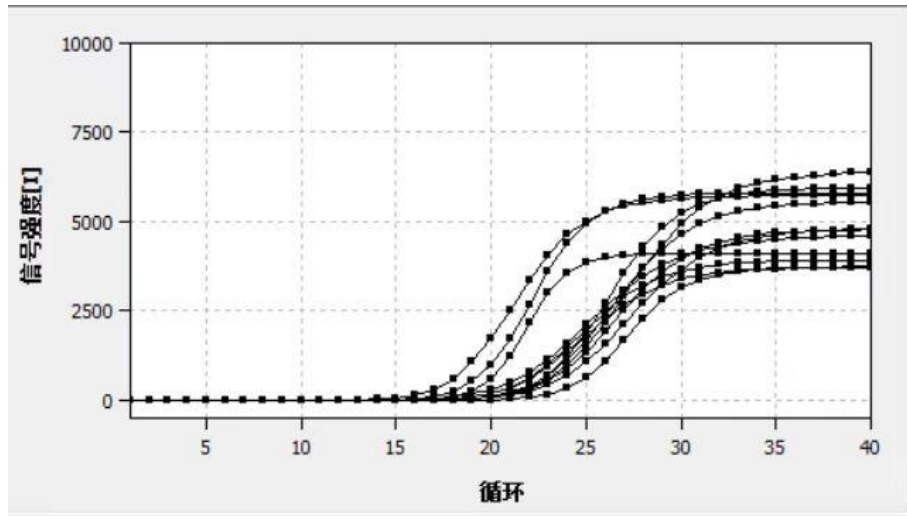

### 2. NFKBIA

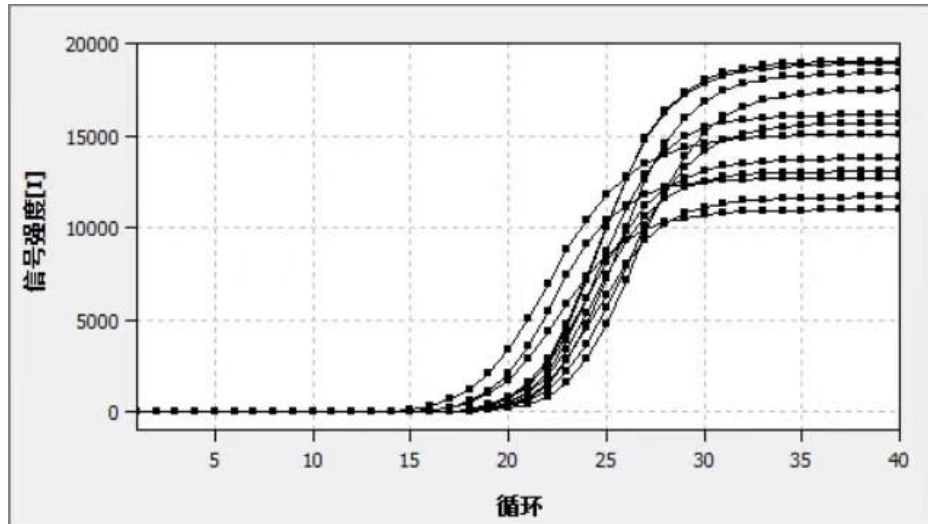

### 3. NFKBIB

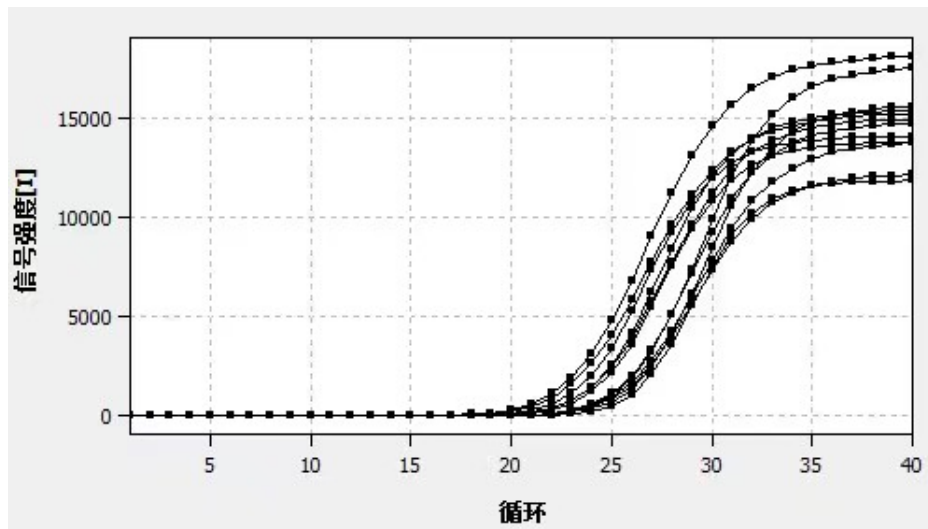

#### 4. NFKBIE

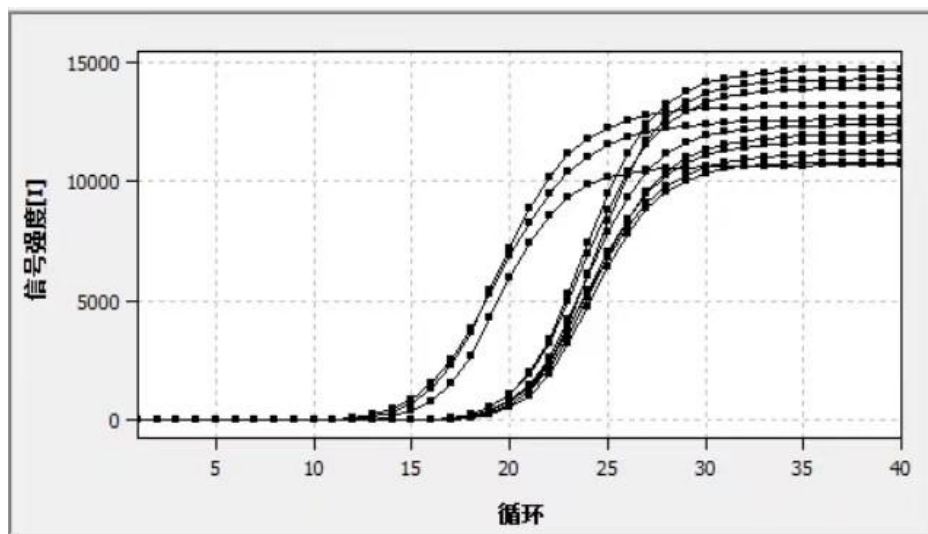

#### 5. IKBKG

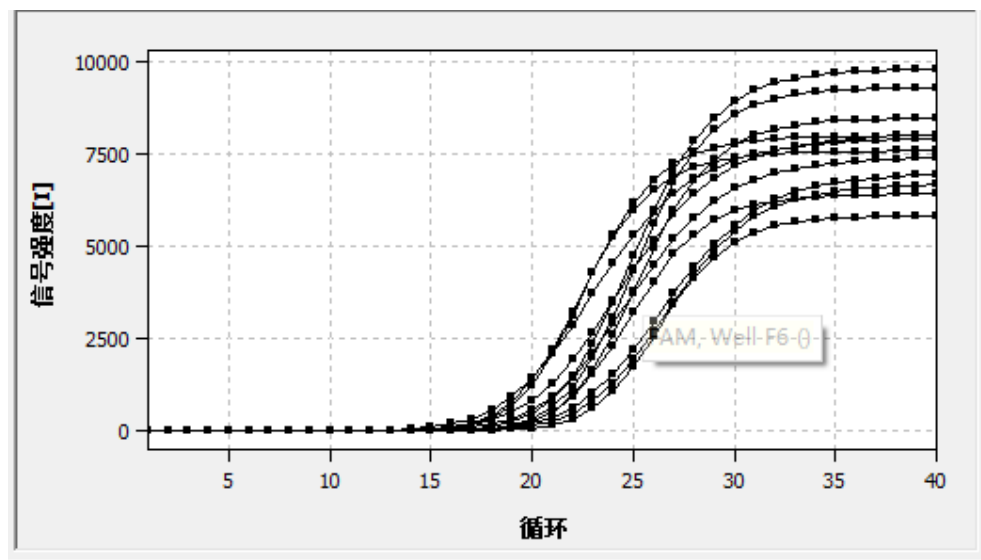

#### 6. NFKBIZ

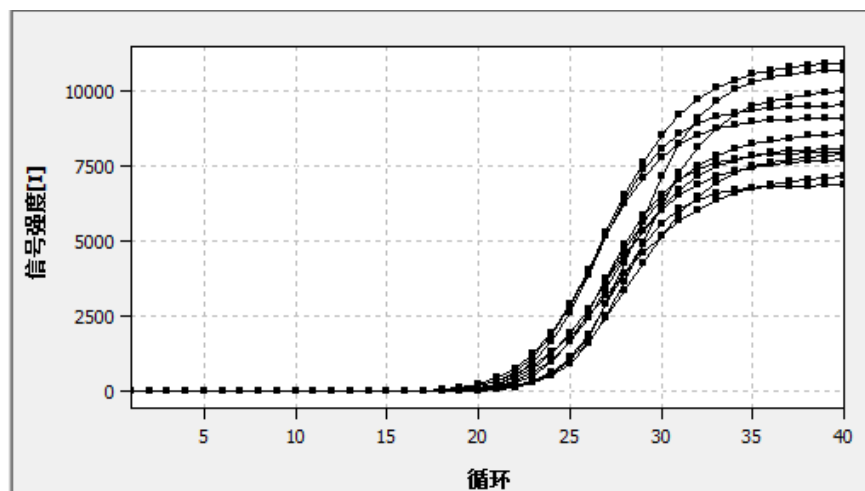

## 7. NFKBID

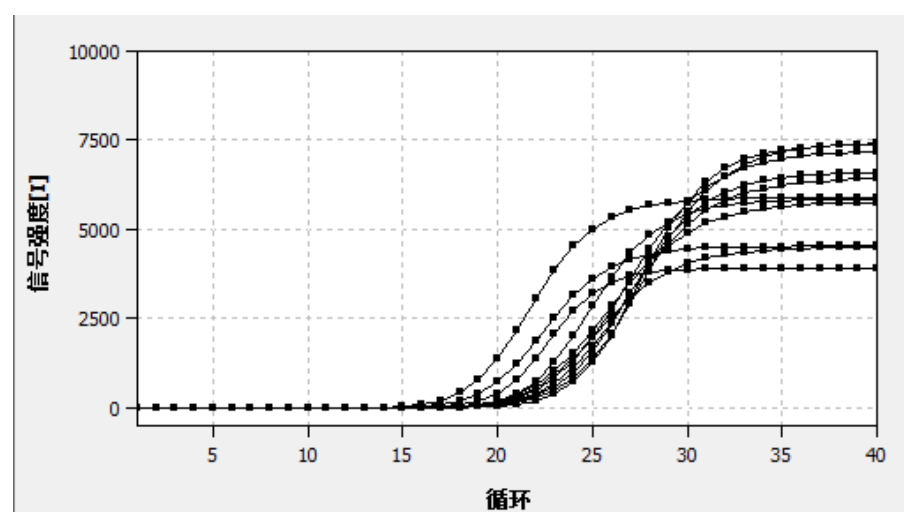

Supplement: Supplementary file 1 [file vetsci-10-00462-s001.zip › Figure S1 Amplification curve.pdf]
